# Blood-Derived Extracellular Vesicle-Associated miR-3182 Detects Non-Small Cell Lung Cancer Patients

**DOI:** 10.3390/cancers14010257

**Published:** 2022-01-05

**Authors:** Kekoolani S. Visan, Richard J. Lobb, Shu Wen Wen, Justin Bedo, Luize G. Lima, Sophie Krumeich, Carlos Palma, Kaltin Ferguson, Ben Green, Colleen Niland, Nicole Cloonan, Peter T. Simpson, Amy E. McCart Reed, Sarah J. Everitt, Michael P. MacManus, Gunter Hartel, Carlos Salomon, Sunil R. Lakhani, David Fielding, Andreas Möller

**Affiliations:** 1 Tumour Microenvironment Laboratory, QIMR Berghofer Medical Research Institute, Herston, QLD 4006, Australia; kekoolani.visan@qimrberghofer.edu.au (K.S.V.); richard.lobb@uq.edu.au (R.J.L.); Luize.GoncalvesLima@qimrberghofer.edu.au (L.G.L.); Sophie.Krumeich@qimrberghofer.edu.au (S.K.); 2 School of Medicine, The University of Queensland, Brisbane, QLD 4006, Australia; 3 Centre for Personalized Nanomedicine, Australian Institute for Bioengineering and Nanotechnology (AIBN), The University of Queensland, Brisbane, QLD 4072, Australia; 4 Centre for Inflammatory Diseases, Department of Medicine, School of Clinical Sciences, Monash University, Clayton, VIC 3168, Australia; shueywen@gmail.com; 5 Bioinformatics Division, The Walter and Eliza Hall Institute of Medical Research, Parkville, VIC 3052, Australia; cu@cua0.org; 6 School of Computing and Information Systems, The University of Melbourne, Parkville, VIC 3010, Australia; 7 Exosome Biology Laboratory, Centre for Clinical Diagnostics, UQ Centre for Clinical Research, Royal Brisbane and Women’s Hospital, Faculty of Medicine, The University of Queensland, Brisbane QLD 4029, Australia; c.palma@uq.edu.au (C.P.); c.salomongallo@uq.edu.au (C.S.); 8 UQ Centre for Clinical Research, The University of Queensland, Brisbane, QLD 4072, Australia; k.ferguson2@uq.edu.au (K.F.); benpgreen78@gmail.com (B.G.); c.niland@uq.edu.au (C.N.); p.simpson@uq.edu.au (P.T.S.); amy.reed@uq.edu.au (A.E.M.R.); s.lakhani@uq.edu.au (S.R.L.); david.fielding@health.qld.gov.au (D.F.); 9 Royal Brisbane and Women’s Hospital, Brisbane, QLD 4029, Australia; 10 Faculty of Science, University of Auckland, Auckland 1010, New Zealand; n.cloonan@auckland.ac.nz; 11 Division of Radiation Oncology, Peter MacCallum Cancer Centre, Melbourne, VIC 3000, Australia; sarah.everitt@petermac.org (S.J.E.); michael.macmanus@petermac.org (M.P.M.); 12 Sir Peter MacCallum Department of Oncology, The University of Melbourne, Parkville, VIC 3010, Australia; 13 Statistics Unit, QIMR Berghofer Medical Research Institute, Herston, QLD 4006, Australia; Hartel@qimrberghofer.edu.au; 14 Departamento de Investigación, Postgrado y Educación Continua (DIPEC), Facultad de Ciencias de la Salud, Universidad del Alba, Santiago 171177, Chile; 15 Pathology Queensland, Royal Brisbane and Women’s Hospital, Brisbane, QLD 4029, Australia; 16 Department of Thoracic Medicine, Royal Brisbane and Women’s Hospital, Brisbane, QLD 4029, Australia

**Keywords:** extracellular vesicles, exosomes, biomarkers, liquid biopsy, miRNA, diagnosis, non-small cell lung cancer

## Abstract

**Simple Summary:**

Lung cancer is the leading cause of cancer-related death worldwide as patients are burdened with incredibly poor prognosis. Low survival rates are primarily attributed to lack of early detection and, therefore, timely therapeutic interventions. Late diagnosis is essentially caused by absent and non-specific symptoms, and compounded by inadequate diagnostic tools. We show here that a lung cancer biomarker, based on a simple blood test, might provide promising advantages for diagnostic assessment. Small extracellular vesicles (sEVs) are miniscule messengers that carry cancer biomarkers and are easily detected in the blood. We identify that the abundance of a specific micro-RNA, miR-3182, in these sEVs can be detected in the blood of lung cancer patients but not in controls with benign lung conditions. This demonstrates the potential use of miR-3182 as a biomarker for lung cancer diagnosis.

**Abstract:**

With five-year survival rates as low as 3%, lung cancer is the most common cause of cancer-related mortality worldwide. The severity of the disease at presentation is accredited to the lack of early detection capacities, resulting in the reliance on low-throughput diagnostic measures, such as tissue biopsy and imaging. Interest in the development and use of liquid biopsies has risen, due to non-invasive sample collection, and the depth of information it can provide on a disease. Small extracellular vesicles (sEVs) as viable liquid biopsies are of particular interest due to their potential as cancer biomarkers. To validate the use of sEVs as cancer biomarkers, we characterised cancer sEVs using miRNA sequencing analysis. We found that miRNA-3182 was highly enriched in sEVs derived from the blood of patients with invasive breast carcinoma and NSCLC. The enrichment of sEV miR-3182 was confirmed in oncogenic, transformed lung cells in comparison to isogenic, untransformed lung cells. Most importantly, miR-3182 can successfully distinguish early-stage NSCLC patients from those with benign lung conditions. Therefore, miR-3182 provides potential to be used for the detection of NSCLC in blood samples, which could result in earlier therapy and thus improved outcomes and survival for patients.

## 1. Introduction

Lung cancer is the leading cause of cancer-related mortality worldwide, predominantly attributed to lack of early detection, timely intervention and appropriate treatment [1,2]. Non-small cell lung cancer (NSCLC) is the most common subtype, comprising 85% of all cases [3], whilst small cell lung cancer (SCLC) primarily accounts for the remaining 15% [4]. Due to lack of early detection, almost 60% of all staged cases of NSCLC patients are diagnosed with advanced, metastatic disease [5,6]. This often makes curative-intent therapies futile in advanced cases [2], as these patients typically experience comorbidities and distant metastases [7]. In addition, aggressive therapies induce adverse events due to severe toxicity, causing premature cessation of treatment [8]. Consequently, therapeutic management in this group is purely palliative [7], hence the five-year survival rate is only 3% [6]. Delayed diagnosis is essentially a result of the asymptomatic nature of early stages, non-specific symptoms of late stages, as well as inadequate diagnostic tools [2]. Currently, the standard methods employed for cancer diagnosis are imaging technologies in conjunction with tissue biopsy [9,10]. However, imaging technologies have limited resolution and provide insufficient information on the lesion [9]. Additionally, there may be risks associated with invasive biopsy procedures, compromising patient health. Inconclusive results generated by these diagnostic methods can lead to prolonged follow-up, contributing to diagnostic inaccuracy [11]. Liquid biopsies potentially provide greater advantages for the diagnosis of cancer as it allows non-invasive, easily accessible sampling and subsequent analysis of cancer biomarkers [10]. Recently, extracellular vesicles (EVs) have emerged as potential candidates for liquid biopsy in cancer detection. Thus the analysis of EVs opening new avenues for diagnosis, prognosis, and treatment outcomes [12].

Small EVs (sEVs) are structures of endocytic origin up to 200 nm in diameter that contain specific cargo reflective of their parental cell [3,13]. SEVs considerably influence intercellular communication as their cargo is transported to recipient cells for uptake, where they alter cell function and physiology, and in the cancer setting, promote cellular transformation into a pro-tumour phenotype [13,14]. In specific circumstances, cancer-derived sEVs are able to induce anti-tumour responses via modulation of the immune system [13]. The lipid bilayer membrane that encases sEV cargo confers high stability of intravesicular contents in the extracellular space, and protection from degradation [15,16]. The secretion of sEVs is an active process performed by all cells of the body and as a result, they are found in bodily fluids such as blood, urine, saliva, breast milk and ascites [10,15,16]. Molecular sEV cargo is composed of an assortment of lipids, proteins, DNA, mRNA and non-coding RNA, such as miRNA, some of which are differentially expressed between normal and cancer cells [5,10]. Notably, sEV miRNAs have critically impacted the field of cancer diagnostics [17,18,19,20]. Cancer-derived sEVs carry functionally active miRNAs [21] which bind target mRNAs to regulate post-transcriptional activity [22,23]. Hence miRNAs packaged in sEVs elicit various effects in recipient cells [17,18,19,20,21]. In the cancer setting, miRNAs function as either oncogenes or tumour suppressors, thus miRNA expression may act as a direct reflection of tumour status. The effect of cancer-derived sEV miRNAs on the manipulation of cellular machinery and subsequent impact on recipient cell function has been reported to promote cancer progression and favour pro-cancer processes [24,25,26,27,28]. Furthermore, sEV miRNAs are protected from RNase activity, thus exhibit increased stability in comparison to circulating, free miRNAs, which makes them ideal targets for establishing cancer biomarkers [29].

Given the lack of specific and sensitive tools for cancer detection, sEV biomarkers would be beneficial for early diagnosis, prognosis, staging, prediction of risk of progression, as well as management of personalised therapy [10,13,17].

We have detected the significant enrichment of miR-3182 in both breast and lung cancer patient blood-derived sEVs, as well as in sEVs derived from lung cancer cells. In a cohort of individuals attending a thoracic hospital unit for diagnosing lung conditions, sEV miR-3182 was capable of distinguishing NSCLC from benign lung tumours with a sensitivity, specificity and accuracy of 84.62%, 78.57% and 80.22%, respectively. This result demonstrates the potential of miR-3182 for clinical applications as a non-invasive biomarker for NSCLC diagnosis.

## 2. Materials and Methods

### 2.1. Patient Sample Collection

This study was approved for the collection and use of all clinical samples by the human research ethics committees from the University of Queensland (2005000785), Royal Brisbane and Women’s Hospital (2005/022 and HREC/18/QRBW/107), Peter MacCallum Cancer Centre (2008001483) and the QIMR Berghofer (P2180). Informed consent was received from all patients. A total of 56 serum samples were collected from patients at the time of primary breast cancer surgery, with 20 benign fibroadenoma and 36 invasive breast carcinoma patients (16 oestrogen receptor positive (ER^+^) and 20 oestrogen receptor negative (ER^−^). NSCLC patient cohort samples were collected as part of the trials at the Peter MacCallum Cancer Centre (ACTRN12611001283965). For the NSCLC detection cohort, patient samples (14 benign lung nodule/s and 12 NSCLC) were collected at the Thoracic unit of the Royal Brisbane and Women’s Hospital (ACTRN12618001789257). Samples were processed within 24 h of collection. Serum was isolated from whole blood by centrifugation at 1200× *g* for 10 min, following which the serum was removed and stored. Plasma was isolated from blood by centrifugation at 1800× *g* for 10 min, following which the plasma was removed and subjected to centrifugation at 1200× *g* for 10 min, then stored. Serum and plasma were snap frozen in 1 mL aliquots and stored at −80 °C until use. All samples were taken prior to the patient receiving therapy. The clinicopathological information of benign fibroadenoma, invasive breast carcinoma and NSCLC patients involved in the analyses is listed in Supplementary Tables S1 and S4.

### 2.2. Cell Culture

Parental HBEC30KT cell line and its oncogenic derivatives (30KT^KRAS^, 30KT^p53/KRAS^, 30KT^p53/EGFR^ and 30KT^p53/KRAS/LKB1^) [30] were maintained in keratinocyte serum-free media (KSFM; Gibco, CA, USA) supplemented with 5 ng/mL human recombinant Epidermal Growth Factor (EGF 1–53] and 50 μg/mL Bovine Pituitary Extract (BPE) (Gibco, CA, USA). All cells were grown at 37 °C with 5% CO_2_. All cell lines were routinely tested for mycoplasma and found to be negative, and their authentication verified using in-house STR profiling.

### 2.3. SEV Isolation

Isolation of sEVs from either patient serum/plasma or cell culture conditioned media was performed by size exclusion chromatography or ultrafiltration, respectively, as previously described [31]. For patient serum/plasma, sEV samples were overlaid on size exclusion columns (Izon Science, Ltd. Christchurch, New Zealand) and eluted with phosphate buffered saline, with sEV-positive fractions collected. The fractions were concentrated in Amicon^®^ Ultra-4 10 kDa centrifugal filter units (Merck Millipore, MA, USA) by centrifugation at 4000× *g* at 4 °C, using an Allegra^®^ X-15R centrifuge (Beckman Coulter, CA, USA). For ultrafiltration, cell culture conditioned media was concentrated with a Centricon Plus-70 Centrifugal Filter (Ultracel-PL Membrane, 100 kDa) device (Merck Millipore, MA, USA), by centrifugation at 3500× *g* at 4 °C, using an Allegra^®^ X-15R centrifuge (Beckman Coulter, CA, USA). Recovery of sEVs was performed with a reverse spin at 1000× *g* for 2 min. The final sEV samples were collected and stored at −80 °C conditions.

### 2.4. Western Blot

**Figure 1:**
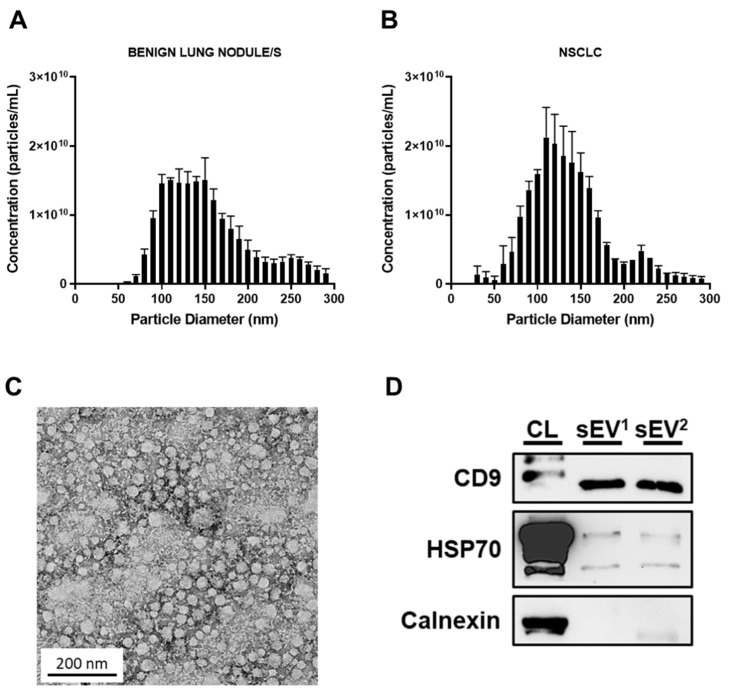
Identification and characterisation of plasma sEVs. (**A**,**B**) Nanoparticle tracking analysis determined the size distribution profiles of isolated sEVs. (**C**) Morphological characterisation of sEVs using transmission electron microscopy. (**D**) Western blot analysis of sEVs (sEV^1^, sEV^2^) derived from lung patient plasma demonstrates the presence of sEV markers, CD9 and HSP70, and the absence of cellular marker, Calnexin. sEV, small extracellular vesicle.

The protein concentrations of cell lysate and sEV samples were quantified by Pierce BCA Protein Assay Kit (Thermofisher, Waltham, MA, USA) and Bradford Assay (Bio-Rad, CA, USA), respectively, and analysed by western blot as previously described [32]. Protein expression was detected using Biorad ChemiDoc Imaging System (Image Lab software version 6.1; Biorad, CA, USA) and enhanced chemiluminescence reagent (ECL Select and Prime; Cytiva, MA, USA) (Figure 1).

### 2.5. Nanoparticle Tracking Analysis

NanoSight NS500 (NanoSight NTA 3.1 Nanoparticle Tracking and Analysis Release Version Build 3.1.46; Malvern Panalytical, Worcestershire, UK) was used according to the manufacturer’s instructions. Samples were processed in duplicate and the camera level set for optimum visualisation to accurately distinguish particles from background noise. Capture videos were analysed to give the mean, mode, and median particle size, as well as an estimate of the number of particles. An Excel spread sheet (Microsoft Corp., Redmond, WA, USA) was automatically generated, showing particle concentrations (Figure 1).

### 2.6. Transmission Electron Microscopy

Transmission electron microscopy was employed for visualisation of sEVs, as previously described [33]. Briefly, sEVs were fixed (3% glutaraldehyde and 2% paraformaldehyde, in cacodylate buffer (pH 7.3)) and applied to a continuous carbon grid and negatively stained. The sEVs were assessed with an FEI Tecnai 12 transmission electron microscope (FEI™, Hillsboro, OR, USA) (Figure 1).

### 2.7. Small RNA Sequencing and Analysis

RNA was extracted from sEVs according to the QIAGEN RNeasy Kit protocol (QIAGEN, Hilden, Germany), with slight modifications. For precipitation, one volume of 70% ethanol was replaced with 1.5 volumes of 100% ethanol. To prevent loss of miRNA before elution, washing Buffer RW1 was replaced with Buffer RWT. RNA QC was performed on a BiOptics QSep and a Thermofisher NanoDrop 2000. SEV RNA was then used to create RNAseq libraries using the NEBNext Small RNA Library Prep Kit (NEB). Libraries were size selected using a Perkin Elmer Labchip XT, and multiplexed for sequencing on an Illumina NextSeq 500 at 1 × 36 bp. FastQ files were aligned to a reference consisting of pre-miRNA hairpins from miRBase v21 (http://miRBase.org, accessed on 7 December 2021). Alignment was performed using RNA-STAR v2.3.0 (reference: http://bioinformatics.oxfordjournals.org/content/29/1/15.full, accessed on 7 December 2021) using the following parameters: “-- runThreadN 16 -- outSAMattributes All -- outFilterMultimapNmax 1”, and all other parameters at default.

### 2.8. SEV miRNA Detection by Quantitative Reverse Transcription-Polymerase Chain Reaction (qRT-PCR)

CDNA template preparation and qRT-PCR was performed using Taqman Advanced miRNA Assays (Thermofisher Scientific, Waltham, MA, USA) according to the manufacturer’s instructions. QRT-PCR was analysed using the ABI Quantstudio 5 System (Thermofisher Scientific, Waltham, MA, USA). All samples were analysed in triplicate. Abundance of miR-3182 was normalised to the housekeeping miRNA, miR-451a. The relative abundance of miR-3182 was calculated using the 2^−∆∆Ct^ method. MiRNA primers were purchased from Life Technologies (Carlsbad, CA, USA).

### 2.9. Statistical Analysis

MiRNA counts were compared between groups (benign fibroadenoma versus invasive breast carcinoma, and invasive breast carcinoma versus NSCLC) using generalised regression with a negative binomial distribution. The fit of the negative binomial distribution to the data was confirmed via QQ plots. The type I error rate for multiple comparisons was controlled using FDR (False Discovery Rate, [34]). The generalised regression was used to calculate a fold-change in miRNA counts between groups for each miRNA. These counts were presented in a volcano plot of −log_10_ FDR-values versus log_2_ fold change. MiRNAs with expected counts of less than 0.5 for both groups were excluded from the analyses. These analyses were done using JMP Pro (v16.0 SAS Institute, Cary, NC, USA).

Statistical analysis for qRT-PCR data was performed using Student’s *t*-test. All experiments were performed in triplicate with a minimum of three independent replicates. Error bars represent mean ± SEM. The area under the curve (AUC) of the receiver operating characteristic (ROC) curve was used to determine the diagnostic value of miR-3182. The optimal cut-off was selected based on the value that yielded the maximal sensitivity and specificity. A *p*-value less than 0.05 was considered statistically significant (* *p* < 0.05; ** *p* < 0.01). Analysis was performed using GraphPad Prism 8.0. 

## 3. Results

### 3.1. Circulating sEV miRNAs Differentiate Invasive Breast Carcinoma from Benign Fibroadenoma, and Identify NSCLC and Oncogenic Transformation of Lung Cells

**Figure 2:**
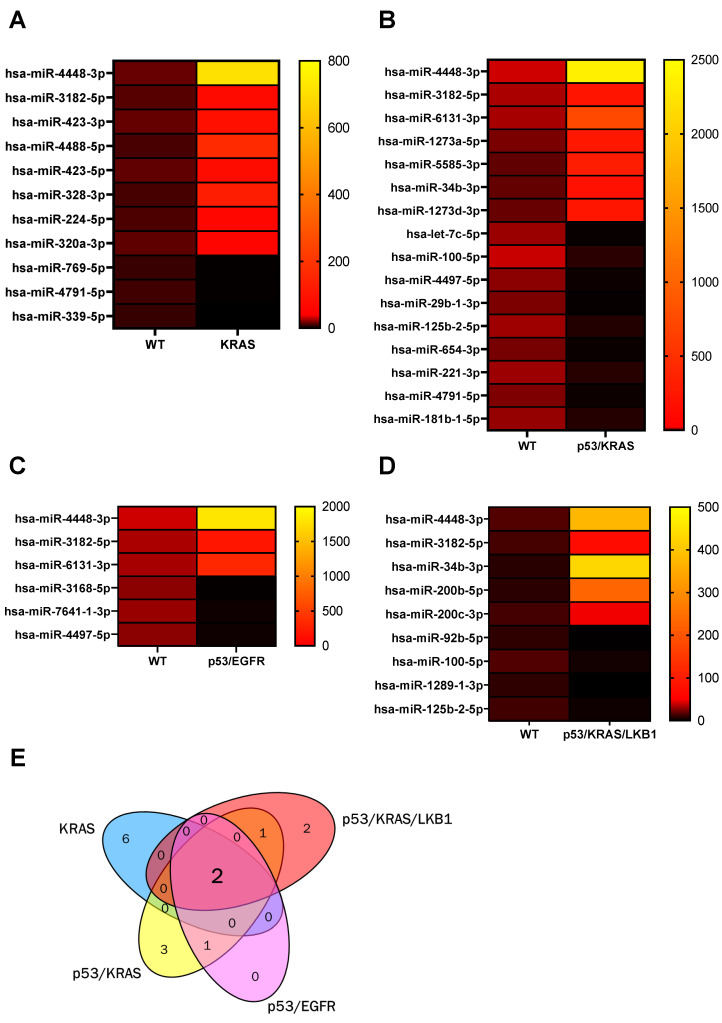
Enrichment of sEV miRNAs is specific to cancer cell mutations. (**A**–**D**) Heat maps demonstrating the average level (log counts per million) of differentially abundant sEV miRNAs derived from oncogene-mutated HBEC lines, HBEC^KRAS^, HBEC^p53/KRAS^, HBEC^p53/EGFR^ and HBEC^p53/KRAS/LKB1^, compared to the untransformed wild-type, HBEC30KT. Higher abundance is denoted by yellow and lower abundance is denoted by black. (**E**) Venn diagram of significantly enriched sEV miRNAs in the oncogene-mutated HBEC lines, compared to the wild type.

The blood of cancer patients contains an amalgamation of sEVs (Figure 1) derived from both normal and cancer cell lineages [35,36,37]. In order to identify sEV-contained miRNAs associated with oncogenic alterations, we compared the miRNA profiles of a human, untransformed bronchial epithelial cell (HBEC) line, HBEC30KT, and four isogenic, oncogenic derivatives. These oncogenic modifications included a single KRAS mutation (HBEC^KRAS^), a dual p53 and KRAS mutation (HBEC^p53/KRAS^), a dual p53 and EGFR mutation (HBEC^p53/EGFR^) and a triple p53, KRAS and LKB1 mutation (HBEC^p53/KRAS/LKB1^). These genetic alterations progress the cell from an untransformed to a transformed phenotype [38]. Using miRNA sequencing, 15 miRNAs were significantly elevated in sEVs derived from the oncogenic transformed cell lines, compared to the wild-type HBEC30KT line (Figure 2). Of the 15 miRNAs, six were enriched only in the HBEC^KRAS^, three only in the HBEC^p53/KRAS^ and two only in the HBEC^p53/KRAS/LKB1^-derived sEVs. Increased abundance of one miRNA, miR-6131, was shared between the HBEC^p53/KRAS^ and HBEC^p53/EGFR^ groups, as well as miR-34b between HBEC^p53/KRAS^ and HBEC^p53/KRAS/LKB1^ groups, in comparison to the wild type. Enriched abundance of only two miRNAs, miR-3182 and miR-4448, was identified in all four oncogenic groups (Figure 2; Supplementary Table S1).

**Figure 3:**
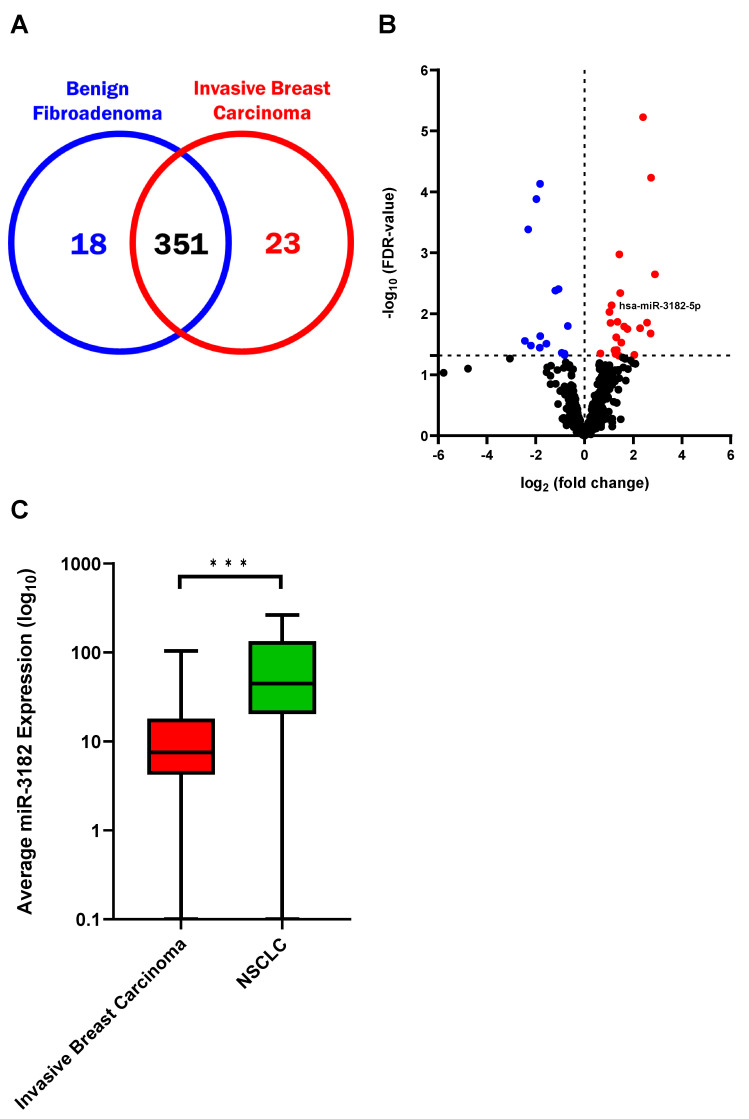
SEV miRNAs distinguish invasive breast carcinoma patients from those with benign fibroadenoma and identify those with NSCLC. (**A**) Venn diagram of a total of 392 miRNAs found in patients with benign fibroadenoma and invasive breast carcinoma. (**B**) Volcano plot of sEV miRNAs identified in the serum of patients with invasive breast carcinoma compared to benign fibroadenoma. The horizontal black dotted line demonstrates the threshold value corresponding to significant FDR-values < 0.05 (Chi-square test). Significantly enriched benign fibroadenoma and invasive breast carcinoma sEV miRNAs are represented as blue and red dots, respectively. (**C**) Box plot of the average abundance (expected counts) of miR-3182 in sEVs derived from invasive breast carcinoma patient serum and NSCLC patient plasma. Boxes range from the first to third quartiles, divided by a line indicating the median (second quartile), with whiskers demonstrating the minimum and maximum. Chi-square test. *** FDR < 0.001.

In order to determine whether any of the sEV miRNAs identified in the oncogenic transformed lung cell lines had clinical significance, particles derived from the blood of benign fibroadenoma and invasive breast carcinoma patients were isolated and purified. Patient sEVs were subjected to miRNA sequencing analysis, which uncovered prominent changes in miRNA expression profiles between benign fibroadenoma and invasive breast carcinoma patients (Supplementary Table S2). Profiling analysis revealed a total of 392 sEV miRNAs contained in the sEVs isolated from the patient samples. Of the 392 miRNAs identified, 351 miRNAs were commonly found in sEVs from both invasive breast carcinoma and benign fibroadenoma serum (Figure 3A,B). Using a stringent cut off (FDR < 0.05 by chi-square analysis), we identified 18 and 23 sEV miRNAs significantly enriched in the serum of benign fibroadenoma and invasive breast carcinoma patients, respectively (Figure 3A,B; Supplementary Table S3). Interestingly, miR-3182, but not miR-4448, was amongst the 23 highly abundant miRNAs in invasive breast carcinoma patient serum.

To investigate whether this panel of 23 highly enriched miRNA was specific for discriminating invasive breast carcinoma, or in fact additionally expressed in other cancer types, miRNA sequencing analysis was performed using sEVs derived from NSCLC patients’ plasma. Comparison of the expression of miR-3182 in invasive breast carcinoma and NSCLC patients revealed miR-3182 to be significantly enriched in NSCLC sEVs (FDR < 0.001, Figure 3C). The abundance of miR-3182 in NSCLC sEVs suggested its suitability as a biomarker of NSCLC detection.

### 3.2. MiR-3182 Distinguishes Early-Stage NSCLC Patients from Subjects with Benign Pathology in Individuals with Lung Nodules Presenting for Investigation

**Figure 4:**
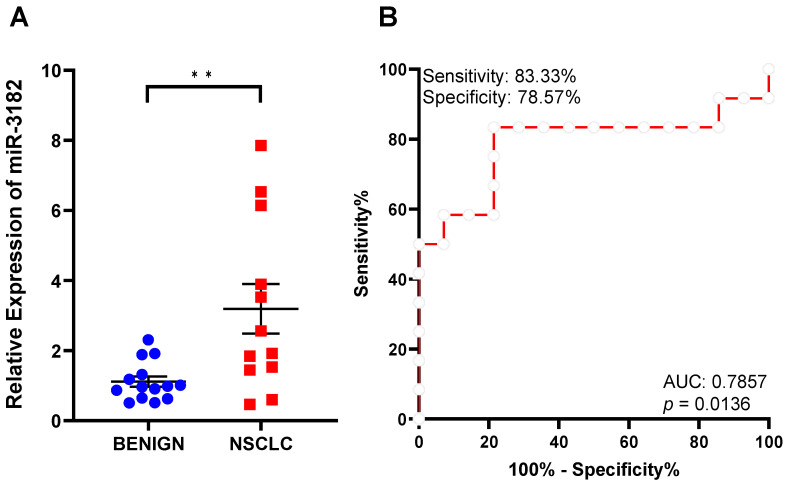
Plasma sEV miR-3182 distinguishes NSCLC patients from patients with benign lung nodule/s. (**A**) Abundance levels (relative expression) of miR-3182 determined by qRT-PCR, in patients with NSCLC compared to those with benign lung nodules. Student’s *t*-test. ** *p* < 0.01. Data are shown as mean ± SEM. (**B**) Receiver operating characteristic (ROC) curve analysis of miR-3182 abundance in benign and NSCLC patient plasma sEVs evaluates the diagnostic capacity of miR-3182.

As miR-3182 presence is specifically elevated in sEVs derived from oncogenic cells, and breast and lung cancer patient blood, we explored the possibility of this miRNA for its diagnostic capacity. From a prospective trial cohort, a total of 27 subjects were used for this analysis, consisting of 12 early-stage NSCLC patients and 14 patients with benign lung nodules or alterations. Factors including age (median of 67.6 and 71.2 years for NSCLC and benign, respectively) and gender were comparable between the two groups (Supplementary Table S4). Patient-derived sEVs were subjected to blind analysis for the abundance of miR-3182. Interestingly, miR-3182 abundance was significantly increased in NSCLC plasma sEVs compared to benign sEVs (*p* < 0.01, Figure 4A). ROC curve analysis determined a sensitivity of 83.33%, specificity of 78.57% and an area under the curve (AUC) value of 0.7857. The 95% confidence interval (CI) is 0.5841 to 0.9873, and a *p*-value of 0.0136 (Figure 4B).

**Figure 5:**
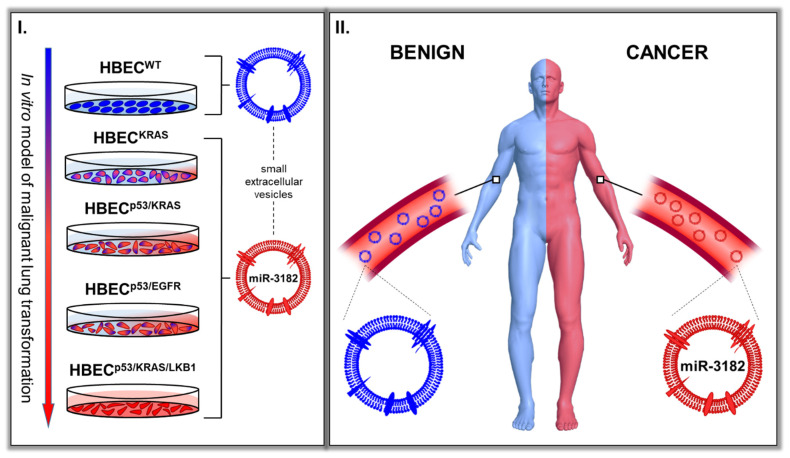
SEV miR-3182 distinguishes, (**I**) transformed cancer cells from untransformed normal cells and (**II**) cancer patients from those with benign tumours.

In summary, our work identified a specific miRNA, miR-3182, that is enriched in sEVs derived from oncogenic, transformed cells and cancer patient blood (Figure 5). Importantly, we showed in a prospective clinical trial, that miR-3182 is capable of distinguishing early-stage NSCLC patients with small tumour burdens from individuals with benign lesions, which currently requires a combination of imaging technologies and tissue biopsies.

## 4. Discussion

SEV miRNAs are a promising prospect of non-invasive biomarkers. The array of sEV miRNAs derived from cancer patients differs significantly from those derived from healthy and benign patients. This distinction suggests utility for screening, early detection, diagnosis, prognosis and treatment prediction [22,39]. Given the heterogeneous nature of the tumour microenvironment, changes in the expression of genetic material are not necessarily reflective of cancer-induced alterations. We have shown that enrichment of sEV miR-3182 is a cancer-specific response, indicating great potential for its diagnostic capacity, which may be purposeful in the clinical field.

Previous literature on the abundance of miR-3182 in cancer-derived sEVs is scarce. Only one other study has reported sEV miR-3182 in normal and malignant lung epithelial cell lines, and patient blood [39]. In contrast to our results, the investigation noted significantly lower levels of miR-3182 in sEVs derived from an NSCLC cell line, A549, compared to a normal, bronchial epithelial cell line, BEAS-2B. No significant difference in sEV miR-3182 between the serum of NSCLC patients and healthy controls was observed [40]. The disparity between the findings of the study and our results may stem from differences in sEV isolation techniques as well as variation in the cell lines used. That is, in our study we employ an isogenic model of lung cancer to profile differences in cancer-derived sEVs. Moreover, age and gender matching between healthy and NSCLC patients was not specified [40], and without appropriate comparison, miRNA expression can be impacted [41].

In accordance with our results, the enrichment of miR-3182 and miR-4448 has also been demonstrated in EVs derived from glioblastoma patient cell lines [42], as well as sEVs derived from hypoxic human primary proximal tubular epithelial cells (PTECs) compared to normal PTECs [43]. Hypoxia is a common phenomenon in malignant tumours that is associated with cancer progression [44], and hypoxic sEVs have been stated to contain miRNAs that promote angiogenesis, migration, invasion and metastasis [45,46,47].

The exact function of miR-3182 has yet to be elucidated, as there are very few publications on its role in normal physiology or cancer. However, it has been suggested that miR-3182 acts as a tumour suppressor [48,49,50,51], as its overexpression in lung cancer, retinoblastoma and osteosarcoma cells resulted in decreased proliferation, invasion, migration and epithelial-to-mesenchymal transition [49,50,51]. Potential function as a tumour suppressor supports the hypothesis that an increase in packaging of miR-3182 into sEVs for extrusion from cancer cells, may contribute to cancer progression. Intriguingly, a previous study analysed expression of plasma miRNAs compared to plasma sEV miRNAs in healthy people. Using qRT-PCR, this study uncovered that there was no significant difference in miR-3182 abundance in plasma or plasma sEVs [52]. It was suggested that miRNAs are distinctively packaged into sEVs in diseased states, as they uncovered higher amounts of oncogenic miRNA in plasma sEVs compared to plasma, in NSCLC patients [52]. This supports the idea that increased packaging of miR-3182 into sEVs is cancer-specific.

Corroborating our findings that sEV miR-3182 abundance is a result of cancer-specific mutations, one study described increased abundance of miR-3182 in sEVs derived from mutant KRAS colorectal cancer cells compared to parental and wild-type KRAS cells [53]. Additionally, increased expression of miR-3182 was reported in mutant EGFR lung adenocarcinoma cell lines, compared to wild-type EGFR lung cancer cell lines [54]. These common cancer mutations seem to have a significant effect on the packaging of miR-3182 into sEVs and intracellular expression. This offers a new perspective to the cell-specific perturbations associated with miRNA expression. Additionally, this suggests potential utility of miR-3182 as a universal cancer biomarker, as mutations of p53, KRAS, EGFR and LKB1 are common amongst a multitude of cancer types [55,56], which we aim to explore further.

Extensive analysis of miR-3182 is required to establish its potential as a marker of prognosis and treatment prediction. Nasopharyngeal carcinoma patients with high levels of miR-3182 correlated with distant metastasis and a worse survival rate, in comparison to those with low miR-3182 abundance [57]. Similarly, miR-3182 was found to be highly expressed in the tissue of patients who died of mantle cell lymphoma compared to patients who did not, suggesting potential as a prognostic marker [58]. The downregulation of miR-3182 in response to treatment has been validated in human epidermoid carcinoma cells that have undergone photodynamic treatment, as well as sorafenib-treated mutant KRAS colorectal cells [59,60]. In an ovarian cancer study, miR-3182 was shown to be downregulated in tissues derived from primary resistant/refractory and chemo-sensitive tumours, compared to normal fallopian tube, as well as in omental metastases compared to normal omentum [61]. Although not in cancer, it is interesting to note that TGF-β1-treatment of human primary fibroblasts resulted in the upregulation of both miR-3182 and miR-4448, compared to untreated fibroblasts, suggesting their involvement in the progression of pulmonary fibrosis [62], a condition associated with an increased risk of developing lung cancer [63]. These data infer that miR-3182 could also function as a biomarker of prognosis and treatment prediction. 

## 5. Conclusions

Overall, our results suggest that sEV miR-3182 could be used as part of a liquid biopsy diagnostic test aimed at screening for cancer in at-risk populations. Validation of miR-3182 as a diagnostic biomarker in a larger cohort of NSCLC patients, as well as in different independent patient cohorts, is instrumental for clinical application. The use of miR-3182 in conjunction with other biomarkers such as cancer-specific sEV proteins to create a biomarker panel would further strengthen its use. Creating a cancer-specific sEV signature would eliminate unnecessary, anachronistic tissue biopsy processes. The implementation of sEV miR-3182 as a diagnostic biomarker could ultimately improve patient outcomes and overall survival.

## Data Availability

The data presented in this study are available in the article and supplementary material.

## Supplementary Materials

The following are available online at https://www.mdpi.com/article/10.3390/cancers14010257/s1, Table S1: Differentially enriched miRNAs in sEVs derived from oncogene-mutated HBEC lines, HBECKRAS, HBECp53/KRAS, HBECp53/EGFR and HBECp53/KRAS/LKB1, compared to the untransformed wild-type, HBEC30KT. HBEC, human bronchial epithelial cell. Table S2: Clinicopathology information of benign fibroadenoma and invasive breast carcinoma patients. ER, oestrogen receptor; PR, progesterone receptor; HER2, human epidermal growth factor receptor 2; TNBC, triple negative breast cancer; DCIS, ductal carcinoma in situ. Table S3: Significantly enriched sEV miRNAs derived from the serum of benign fibroadenoma and invasive breast carcinoma patients. Table S4: Clinicopathology information of patients with benign lung nodule/s and NSCLC. NSCLC, non-small cell lung cancer; TNM, tumour, node, metastasis.
