# Supplementary material for: Blood-Derived Extracellular Vesicle-Associated miR-3182 Detects Non-Small Cell Lung Cancer Patients
Source: Cancers (Basel). 2022 Jan 5;14(1):257. doi: 10.3390/cancers14010257 (PMC8750562; doi:10.3390/cancers14010257)
Supplement: Supplementary file 1 [file cancers-14-00257-s001.zip › cancers-14-00257-s001/cancers-1521035-supplementary.pdf]

HBEC Line	Enriched miRNAs	*p*-value	Reduced miRNAs	*p*-value
**KRAS**	hsa-miR-4448-3p	2.91E-09	hsa-miR-769-5p	2.68E-04
	hsa-miR-423-3p	7.13E-05	hsa-miR-4791-5p	3.89E-04
	hsa-miR-4488-5p	7.23E-05	hsa-miR-339-5p	6.80E-04
	hsa-miR-423-5p	2.88E-04		
	hsa-miR-328-3p	3.79E-04		
	hsa-miR-3182-5p	6.33E-04		
	hsa-miR-224-5p	9.42E-04		
	hsa-miR-320a-3p	1.08E-03		
**p53/KRAS**	hsa-miR-4448-3p	4.65E-13	hsa-let-7c-5p	5.52E-06
	hsa-miR-6131-3p	1.75E-08	hsa-miR-100-5p	2.29E-05
	hsa-miR-3182-5p	3.21E-08	hsa-miR-4497-5p	4.82E-05
	hsa-miR-1273a-5p	9.14E-05	hsa-miR-29b-1-3p	2.64E-04
	hsa-miR-5585-3p	4.73E-04	hsa-miR-125b-2-5p	3.00E-04
	hsa-miR-34b-3p	7.70E-04	hsa-miR-654-3p	1.09E-03
	hsa-miR-1273d-3p	9.59E-04	hsa-miR-221-3p	1.12E-03
			hsa-miR-4791-5p	1.40E-03
			hsa-miR-181b-1-5p	1.43E-03
**p53/EGFR**	hsa-miR-4448-3p	4.49E-12	hsa-miR-3168-5p	1.31E-05
	hsa-miR-3182-5p	3.10E-07	hsa-miR-7641-1-3p	4.92E-05
	hsa-miR-6131-3p	3.92E-06	hsa-miR-4497-5p	1.15E-04
**p53/KRAS/LKB1**	hsa-miR-4448-3p	2.34E-07	hsa-miR-92b-5p	1.52E-05
	hsa-miR-34b-3p	1.55E-04	hsa-miR-100-5p	9.19E-05
	hsa-miR-3182-5p	4.35E-04	hsa-miR-1289-1-3p	2.38E-04
	hsa-miR-200b-5p	5.63E-04	hsa-miR-125b-2-5p	3.79E-04
	hsa-miR-200c-3p	8.34E-04		

Visan et al. Supplementary Table S1

	Invasive Breast Carcinoma		Benign Fibroadenoma
	ER^+	ER^-	
**Patient Age at Collection (years)**			
< 40	2	3	14
40-60	8	8	5
60-80	4	9	1
> 80	2	-	-
Median	54.9	59.2	33.7
**Tumour Subtype**			
ER^+	16	-	NA
PR^+	12	2	NA
$HER2^+$	6	1	NA
TNBC	-	17	NA
**Tumour Size (mm)**			
Range	12-90	3-80*	NA
Median	25	18	NA
**Tumour Grade (n)**			
1	-	1	NA
2	-	1	NA
3	16	18	NA
**In Situ Disease (DCIS) (n)**	11	13	NA
**Lymphovascular Invasion (n)**	3	3	NA
**Multifocality (n)**	3	3	NA
**Multicentricity (n)**	2	1	NA
**Lymph Node (n)**			
Positive	9	8	NA
Negative	7	12	NA

* Incomplete information available

Visan et al. Supplementary Table S2

Benign Fibroadenoma	FDR-value	Invasive Breast Carcinoma	FDR-value
hsa-miR-7-2-5p	7.34E-05	hsa-miR-7641-1-3p	5.92E-06
hsa-miR-7-1-5p	7.38E-05	hsa-miR-375-3p	5.83E-05
hsa-miR-7-3-5p	7.39E-05	hsa-miR-7641-2-3p	1.06E-03
hsa-miR-1-1-3p	1.31E-04	hsa-miR-6131-3p	2.24E-03
hsa-miR-1-2-3p	1.31E-04	hsa-miR-423-5p	4.57E-03
hsa-miR-184-3p	4.12E-04	hsa-miR-3182-5p	7.22E-03
hsa-miR-224-5p	3.93E-03	hsa-miR-146a-5p	9.32E-03
hsa-miR-1246-5p	4.15E-03	hsa-miR-409-3p	1.36E-02
hsa-miR-27a-3p	1.58E-02	hsa-miR-4433b-3p	1.39E-02
hsa-miR-130b-3p	2.32E-02	hsa-miR-320a-3p	1.41E-02
hsa-miR-105-1-5p	2.78E-02	hsa-miR-4488-5p	1.61E-02
hsa-miR-105-2-5p	2.78E-02	hsa-miR-4492-3p	1.72E-02
hsa-miR-582-3p	3.10E-02	hsa-miR-625-3p	1.77E-02
hsa-miR-204-5p	3.31E-02	hsa-miR-1273h-3p	2.10E-02
hsa-miR-454-3p	3.61E-02	hsa-miR-328-3p	2.43E-02
hsa-miR-31-5p	4.36E-02	hsa-miR-654-3p	2.96E-02
hsa-miR-365a-3p	4.50E-02	hsa-miR-3960-3p	3.93E-02
hsa-miR-365b-3p	4.73E-02	hsa-miR-3615-3p	3.98E-02
		hsa-miR-148a-3p	4.47E-02
		hsa-miR-148a-5p	4.53E-02
		hsa-miR-3168-5p	4.59E-02
		hsa-miR-505-5p	4.72E-02
		hsa-miR-136-3p	4.85E-02

Visan et al. Supplementary Table S3

		NSCLC	Benign
**Patient Age at Collection (years)**			
	40-60	2	4
	60-80	9	10
	>80	1	-
**Gender**			
	Male	6	7
	Female	6	7
**Tumour Stage (TNM)**			
	IA1	1	NA
	IA2	3	NA
	IA3	1	NA
	IB	6	NA
	IIIA	1	NA
**Lymph Node Status**			
	N0	11	NA
	N1	-	NA
	N2	1	NA
