# Supplementary figures and images for: Blood-Derived Extracellular Vesicle-Associated miR-3182 Detects Non-Small Cell Lung Cancer Patients
Source: Cancers (Basel). 2022 Jan 5;14(1):257. doi: 10.3390/cancers14010257 (PMC8750562; doi:10.3390/cancers14010257)

**Calnexin**

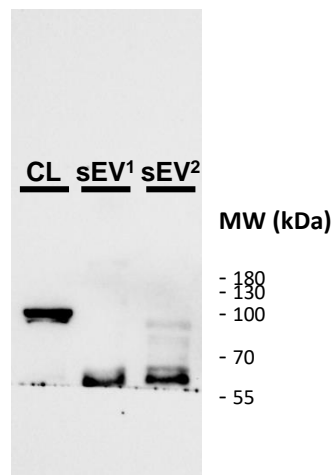

**HSP70**

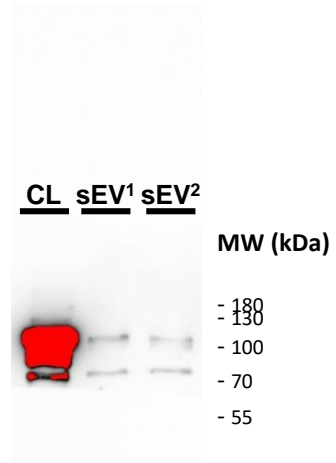

**$\alpha$  CD9**

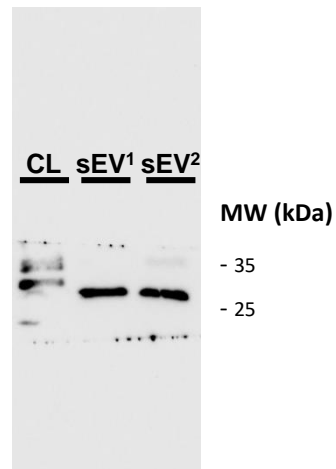

Supplement: Supplementary file 1 [file cancers-14-00257-s001.zip › cancers-14-00257-s001/cancers-1521035-original-images.pdf]
